# Unveiling the Effect of Magnetic Noise in the Coherence of Single-Molecule Quantum Processors

**DOI:** 10.3389/fchem.2019.00662

**Published:** 2019-10-01

**Authors:** Luis Escalera-Moreno, José J. Baldoví

**Affiliations:** ^1^Instituto de Ciencia Molecular, Universidad de Valencia, Paterna, Spain; ^2^Max Planck Institute for the Structure and Dynamics of Matter, Hamburg, Germany

**Keywords:** coordination chemistry, polyoxometalate, molecular magnetism, molecular nanomagnet, molecular spin qubits, decoherence, scalability, quantum algorithm

## Abstract

Quantum bits (qubits) constitute the most elementary building-blocks of any quantum technology, where information is stored and processed in the form of quantum superpositions between discrete energy levels. In particular, the fabrication of quantum processors is a key long-term goal that will allow us conducting specific tasks much more efficiently than the most powerful classical computers can do. Motivated by recent experiments in which three addressable spin qubits are defined on a potential single-molecule quantum processor, namely the [Gd(H_2_O)P_5_W_30_O_110_]^12−^ polyoxometalate, we investigate the decohering effect of magnetic noise on the encoded quantum information. Our state-of-the-art model, which provides more accurate results than previous estimates, show a noticeable contribution of magnetic noise in limiting the survival timescale of the qubits. Yet, our results suggest that it might not be the only dephasing mechanism at play but other mechanisms, such as lattice vibrations and physical movement of magnetic nuclei, must be considered to understand the whole decoherence process.

## Introduction

The most basic component for quantum information storage and processing is the so-called qubit, the quantum version of the bit (Nielsen and Chuang, 2010). The combination of several entangled qubits–generally, two or three–gives rise to a logical gate, the most elementary unit in any quantum algorithm devoted to execute a specific task. Indeed, an algorithm is nothing but a sequence of logical gates. The possibility of developing more sophisticated algorithms, hence involving a larger number of logical gates, critically depends on our ability to entangle an increasing number of qubits without damaging the encoded information. This is what is known as scalability. For this purpose, it is crucial to control both the position and the orientation of the relevant physical qubits in space so that they can properly communicate with each other.

As far as this spatial organization is concerned, molecular spin qubits (MSQ) are especially promising (Ding et al., 2016; Gaita-Ariño et al., 2019). These qubits are encoded in the spin energy levels of magnetic coordination compounds, which commonly consist of a single magnetic metal ion coordinated by a set of ligands. It is precisely because of the versatility offered by synthetic chemistry what confers upon these MSQs the important property of being tailored at will with the desired features. There already exist some proposals of spatial organization based on (i) binding-tag biomolecules, where the spin qubits are communicated via magnetic dipolar interaction (Gaita-Ariño et al., 2016; Rosaleny et al., 2018), and (ii) templated 2D arrays where the exchange of information between the qubits is mediated by a net of waveguides (Jenkins et al., 2016).

**Figure 1:**
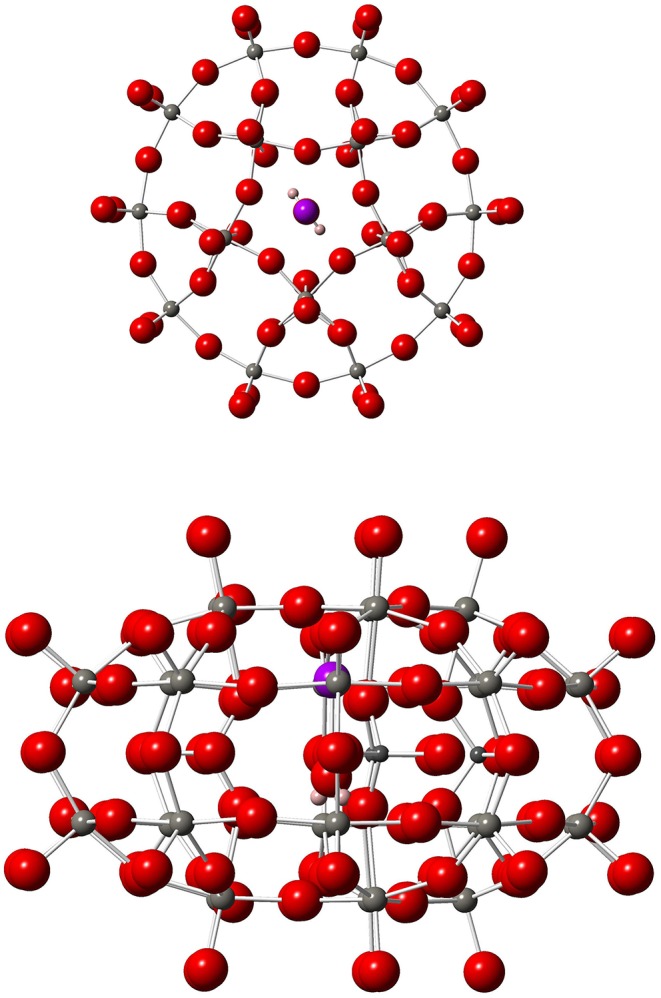
Top (left) and side (right) views of **1**. Gd, Purple; W, medium gray; P, dark gray; O, red; H, pastel orange.

**Figure 2:**
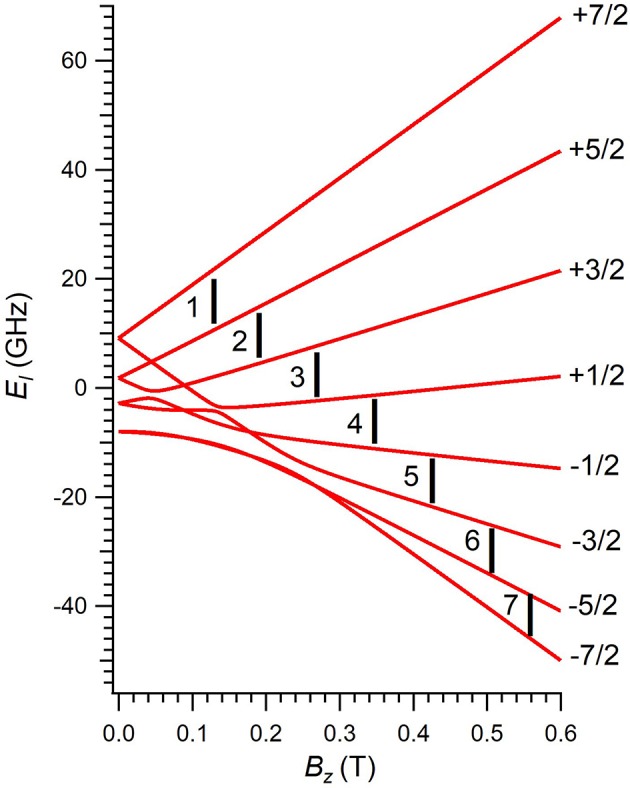
Zeeman energy scheme of **1** as a function of the applied magnetic field, derived by diagonalizing Equation (1). Each Zeeman curve is labeled with the corresponding *m*_*S*_ projection of the ground electron quantum number *S* = 7/2 of Gd^3+^. Numbers 1–7 label each resonant transition with the working microwave frequency.

An alternative strategy to spatially arrange many copies of a given spin qubit is to design a magnetic molecule whose energy scheme allows defining more than one qubit in it. The implementation of *n* qubits requires 2^*n*^ isolated energy levels and, importantly, the transition between each pair of energy levels must be addressable and distinguishable from the rest. Thus, it would be possible to perform a given quantum algorithm by using a single-molecule. In other words, the molecule itself becomes an autonomous and independent quantum processor. This is precisely what has recently been proposed on a Gd^III^-based magnetic polyoxometalate (POM), namely [Gd(H_2_O)P_5_W_30_O_110_]^12−^, hereafter referred to as **1** (Jenkins et al., 2017). This molecule consists in a single Gd^III^ ion entrapped inside a POM cage, whose ligand distribution (ten oxygen atoms and one apical water molecule H_2_O) results in a 5-fold geometry around the magnetic ion, see Figure 1. The Gd-O distance between Gd^III^ and the oxygen atom of H_2_O is 2.259 Å, which is compatible with a coordination bond. Its low-lying energy scheme consists in the eight isolated levels of the ground *J* = 7/2 multiplet, see Figure 2, and thus allows defining three qubits (*n* = 3) that can be entangled by means of Electron Paramagnetic Resonance (EPR) spectroscopy (Schweiger and Jeschke, 2001; Baldoví et al., 2013a). In this technique, microwave radiation pulses of a precise length are used to drive transitions between pairs of spin energy levels. A quantum algorithm is thus performed by a proper pulse sequence. As a matter of fact, there exist simple algorithms exactly involving three qubits, which could be implemented on **1** such as one of the Shor's quantum error correction codes (Shor, 1995; Baldoví et al., 2015).

Of course, the implementation of more and more complex algorithms will require the interplay of an increasing number of qubits. In the case of **1**, this would generally mean to assemble several copies in close enough positions to allow qubit entanglement. Nonetheless, approaching magnetic molecules with the goal of communicating them also leads to an unavoidable detrimental effect. Indeed, the nature of the involved molecular entities produces a magnetic field that is permanently covering all the space. This field results in a magnetic noise which will eventually end up destroying the information at play during the algorithm. This is what is known as decoherence (Nielsen and Chuang, 2010), and one of the most important current goals is to design MSQs as robust as possible against it (Ding et al., 2016).

The successful implementation of **1** in a device devoted to perform generic quantum algorithms will require close enough copies of **1** to allow inter-qubit communication, but with a weak enough magnetic noise to keep quantum information -either stored or under processing- safe. The timescale in which this information -encoded in the form of superpositions between pairs of energy levels- is safe from decoherence is characterized by the so-called phase memory time *T*_*m*_. This characteristic time can be determined by means of EPR experiments (Schweiger and Jeschke, 2001), and of course, should be larger than the execution time of the algorithm. In these experiments, the in-plane magnetization decay *M*_⊥_(*t*) of the sample is measured as a function of time. The phase memory time *T*_*m*_ is routinely extracted by fitting the plot *M*_⊥_(*t*) vs. *t* to an exponential function of the form exp(−*t*/*T*_*m*_). Thus, one of the current key goals is to design magnetic molecules with long enough phase memory times.

In this manuscript, we aim to calculate the effect of magnetic noise –present in a single crystal of many equally-oriented copies of **1** at a given concentration– to check whether it is the decoherence source limiting the *T*_*m*_ of a given set of pairs of energy levels in **1**. An estimate yet rough was already provided in Jenkins et al. (2017); we will employ a state-of-the-art model recently developed by some of us (Escalera-Moreno et al., 2019). While previous models failed, ours succeeded at reproducing the experimental *T*_*m*_ in challenging systems where magnetic noise is the limiting decoherence source (Shiddiq et al., 2016). The particular set of pairs of energy levels is composed of those pairs whose transitions are resonant with the microwave frequencies employed in the so-called X-band of an EPR spectrometer, which range between ~9–10 GHz (Schweiger and Jeschke, 2001). We will compare our calculated values denoted as $T_{m}^{e}$ with the experimental ones of **1**, and discuss on the presence of other decoherence sources that may also be limiting *T*_*m*_.

## Methods

First of all, one needs to determine the electronic structure of **1**, namely, energies *E*_*l*_ and wave-functions |ψ_*l*_〉 of the magnetic level scheme. In the case of a single magnetic metal ion coordinated by a set of donor atoms this is accomplished by diagonalizing the Crystal Field Hamiltonian Ĥ which, for **1**, takes the particular expression in Equation (1):

(1) $$\begin{array}{r} Ĥ=D\left[{Ŝ}_{z}^{2}-\frac{1}{3}S\left(S+1\right)\right]+E\left({Ŝ}_{x}^{2}-{Ŝ}_{y}^{2}\right)+\mu_{B}g\vec{B}\cdot \vec{S} \end{array}$$

In Equation (1), the crystal field generated by the coordinating atoms is described by the first two terms, and its effect is to lift the degeneracy of the 2*S* + 1 = 8 energy levels corresponding to the ground electron spin quantum number *S* = 7/2 of the Gd^III^ ion. The eight energies are parametrized as a function of the axial *D* and rhombic *E* zero-field anisotropy parameters, whose values *D* = 1,281 MHz, *E* = 294 MHz were determined by means of EPR spectroscopy (Jenkins et al., 2017). Ŝ_*x*_, Ŝ_*y*_, Ŝ_*z*_ are the cartesian components of the electron spin operator Ŝ. The change in the energies of the eight magnetic levels due to the magnetic field $\vec{B}$ -with a fixed direction- applied by the EPR spectrometer is introduced via a Zeeman term in Equation (1). μ_*B*_ is the Bohr magneton, and *g* = 2 is the free-ion value for the electron Landé factor *g* of the Gd^III^ ion. The diagonalization of the Crystal Field Hamiltonian in Equation (1) is performed by employing the computational package SIMPRE (Baldoví et al., 2013b, 2014).

Our theoretical estimate $T_{m}^{e}$ of *T*_*m*_ for each given pair of energy levels is based on a model recently developed by some of us. The full details on the model derivation are rather tedious and extensive for the present manuscript, but can be found elsewhere by the readership interested either in reproducing our results or in studying other molecular systems (Escalera-Moreno et al., 2019). The $T_{m}^{e}$ expression is given in Equation (2):

(2) $$\begin{array}{r} T_{m}^{e}=\frac{2\hbar }{\Delta \gamma_{e}} \end{array}$$

where ℏ is the reduced Planck's constant, Δ is the energy gap between the two given magnetic levels, and γ_*e*_ is the electron dephasing rate. This rate depends on Δ, *g*, the working temperature *T*, the 2*S* + 1 energies *E*_*l*_, the expectation values of the squared electron spin operator components ${\left\{\langle \psi_{l}|{Ŝ}_{\alpha }^{2}|\psi_{l}\rangle \right\}}_{l=1,\ldots ,2S+1;\alpha =x,y,z}$, and the cartesian positions of the Gd^III^ ions in the single crystal. We use *g* = 2 and the value of the experimental temperature *T* = 6 K.

The cartesian positions of the Gd^III^ ions in the single crystal were determined by X-ray crystallography (Jenkins et al., 2017). Since the single crystal used in the EPR experiments contains a number of Gd^III^ ions comparable to an Avogadro's number, which is too large to be handled in a computer, we extract several spherical clusters with increasing radii of hundreds of Å from the single crystal. For each given pair of energy levels, we calculated γ_*e*_ at each one of these clusters and found that, for a large enough radius, γ_*e*_ converges to a limit value. This means that including additional Gd^III^ ions beyond the converging cluster radius does not produce any effect on the calculated γ_*e*_ anymore, and hence proves that there is no need to deal with the whole single crystal. A radius of 400 Å was enough to converge the calculated γ_*e*_ in each pair of energy levels explored.

The simulation of a given concentration below 100% implies to remove a proper number of Gd^III^ from the spherical cluster before calculating γ_*e*_. Since the working concentration of the single crystal employed in the EPR experiments was 1%, this means that we need to remove 99 out of 100 Gd^III^ ions. For that purpose, we sweep all Gd^III^ ions and at each one we generate a random number *x* between 0 and 1. If 0 ≤ *x* < 0.01, we save the ion position; otherwise, we drop it. This results in a random dilution that matches the desired concentration. Nonetheless, there does not exist of course a unique way to randomly distribute the Gd^III^ ions in the cluster to produce the given concentration. We thus generate several random dilutions all of them with the same concentration of 1%, and calculate γ_*e*_ at each one. Since all of these dilutions are equally likely to occur, we use the arithmetic mean $\bar{\gamma_{e}}$ to determine $T_{m}^{e}$ as $T_{m}^{e}=2\hbar /\Delta \bar{\gamma_{e}}$.

A last important point is the direction of the applied magnetic field $\vec{B}$. The orientation of the single crystal in the EPR spectrometer was such that this direction coincides with the perpendicular axis to the molecular plane of **1**, see Figure 1 (left), which defines the *z* axis direction. Thus, when calculating γ_*e*_ we apply the magnetic field in this perpendicular axis which is common to all copies of **1** in the crystal since all of them are equally oriented. Hereafter, the magnitude $\left|\vec{B}\right|$ of the applied magnetic field will be referred to as *B*_*z*_.

## Results and Discussion

The diagonalization of Ĥ in Equation (1) provides the eight energies *E*_*l*_ of the ground *S* = 7/2 multiplet of **1** as a function of *B*_*z*_, see Figure 2. Despite Gd^III^ ion has a ground *S* = 7/2 multiplet with a ground orbital angular momentum *L* = 0, there exists a non-zero overall energy splitting (~20 GHz) at *B*_*z*_ = 0. As expected from an ion whose contribution to the ground angular momentum is purely spin, **1** should be rather insensitive to the ligand environment. This would result in an isotropic low-lying Zeeman energy scheme independent of the field direction, with eight degenerate energies at *B*_*z*_ = 0.

The reason behind the overall energy splitting at zero field, i.e., the loss of isotropy, is found in the mixing between ground and excited multiplets with *L* ≠ 0. Indeed, the first-principles treatment of **1**, which consists in solving a Schrödinger-like equation where all multiplets are included into the so-called full Hamiltonian, would reveal that the lowest eight wave-functions depend on both ground and excited multiplets. In Equation (1), we are only considering the ground multiplet, whereas all the effects derived from its interaction with excited multiplets come effectively encoded in *D* and *E*. This is what defines the Crystal Field Approximation, where the low-lying energy level scheme of the full Hamiltonian is projected onto an effective spin Hamiltonian which depends only on spin degrees of freedom, while the effects derived from spin-orbit coupling are encoded in the anisotropy parameters. Note that in case of **1** the zero-field energy splitting is much smaller as compared to similar compounds with a different lanthanide ion (Shiddiq et al., 2016). Thus, excited multiplets might be high enough in energy to assume that the lowest energy levels are well-isolated and hence safely define three qubits.

**Figure 3:**
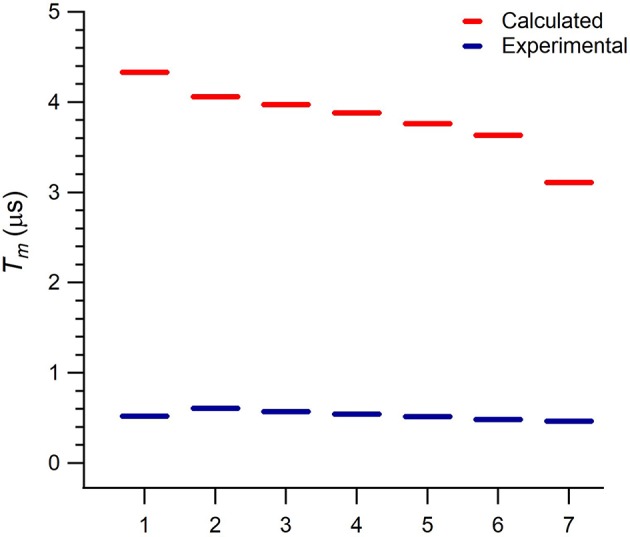
Calculated and experimental phase memory times *T*_*m*_ of **1** at each one of the seven resonant transitions in the explored magnetic field range (1: 0.125 T, 2: 0.19 T, 3: 0.27 T, 4: 0.35 T, 5: 0.43 T, 6: 0.51 T, 7: 0.56 T).

The experimental implementation of **1** as a three-qubit quantum processor would require to choose a fixed magnetic field such that each transition is distinguishable from the rest. Thus, the execution of a given algorithm will need to apply pulses of many different frequencies, which could be achieved by an Arbitrary Waveform Generator. Herein, we have explored the magnetic field range 0.0–0.6 T, and the transitions we aim to study are those whose driving energies Δ are inside the frequency range 9–10 GHz, see Figure 2. Note that Equation (2) would also be valid at any other driving energies, we select these magnetic field and frequency ranges as they are of common use in EPR spectroscopy. The comparison between calculated $T_{m}^{e}$ and experimental *T*_*m*_ values for these particular transitions is depicted in Figure 3.

A noticeable feature in Figure 3 is the monotonous decrease in the calculated $T_{m}^{e}$. Indeed, this is because $E_{e}^{2}$ is constant, while Δ monotonously decreases from the first to the seventh transition. This behavior is also observed in the experimental *T*_*m*_, although with a slighter decrease and with the value of the first transition lying out of this trend possibly because of a larger experimental error. The calculated $T_{m}^{e}$ values are around 3 μs above the experimentally reported *T*_*m*_ values. Unlike the rough estimate provided in Jenkins et al. (2017), our results suggest that electron magnetic noise is not the limiting source of decoherence or, because of the proximity between $T_{m}^{e}$ and *T*_*m*_, may not be the only mechanism limiting *T*_*m*_.

To check whether electron magnetic noise is among the mechanisms limiting *T*_*m*_, it is useful to measure *T*_*m*_ at different Gd^III^ ion concentrations as did in Martínez-Pérez et al. (2012). These measurements revealed that indeed *T*_*m*_ decreases as Gd^III^ ion concentration increases, which means that electron magnetic noise might be one of the decoherence mechanisms limiting *T*_*m*_. Nonetheless, it might not be the only one in the light of the two following reasons. First, as mentioned above, the theoretical estimates $T_{m}^{e}$ do not match the experimental *T*_*m*_ values. Second, *T*_*m*_ does not scale with the Gd^III^ ion concentration *x* as *T*_*m*_ ∝ 1/*x*. This scaling characterizes electron magnetic noise as the only decoherence mechanism limiting *T*_*m*_ and, in this case, establishes that a rise in *x* of one order of magnitude should divide *T*_*m*_ by 10 (Escalera-Moreno et al., 2019; Lunghi and Sanvito, 2019).

A well-established strategy to reduce the effect of electron magnetic noise is by further dilution of the given single crystal. Nonetheless, this is not a useful method since it implies to move the Gd^III^ ions away, which hence can result in an important alteration of the inter-qubit communication. Thus, one needs to choose a concentration that allows a robust qubit communication while keeping electron magnetic noise below a safe threshold. In a 100% concentrated single crystal of **1** the minimum distance between pairs of Gd^III^ ions is around 17 Å, while in our 1% working concentration this minimum distance is increased up to ~37 Å. If this distance is still too large and further approach of Gd^III^ ions is required to allow a safe inter-qubit communication, one should first identify and suppress the other decoherence sources before rising the single crystal concentration.

**Figure 4:**
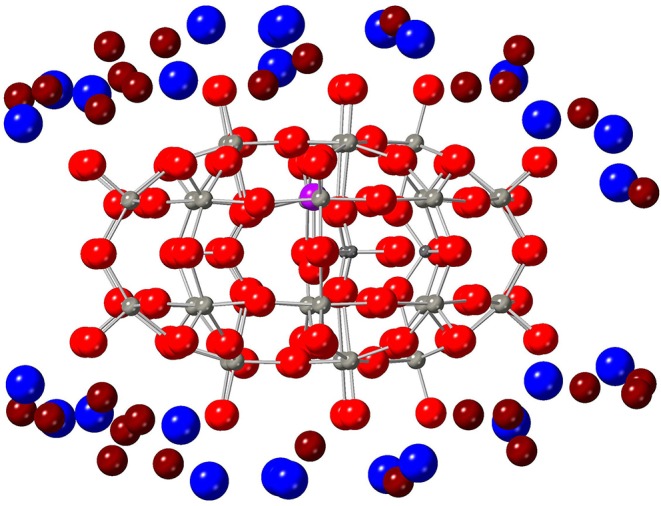
View of **1** surrounded by the crystallographic positions of some K cations (blue) and water molecules (O atoms in dark red, H atoms omitted for clarity).

Let us recall that **1** is a charged molecule, which requires the presence of counter-ions to properly balance the overall charge. Thus, the single crystal is composed of several copies of **1** surrounded by water molecules and potassium cations acting as counter-ions, see Figure 4. Importantly, the single crystal is composed of chemical elements that present a high occurrence of isotopes with magnetic nuclei. Namely, tungsten (14%, *I* = 1/2) and phosphorus (100%, *I* = 1/2) atoms in the polyoxometalate cage, hydrogen atoms (100%, *I* = 1/2) in water molecules, and potassium cations (100%, *I* = 3/2), where *I* is the isotope nuclear spin quantum number. This bath of magnetic nuclei generates an extra magnetic noise which adds to that of the Gd^III^ ion bath and, of course, can also affect quantum information, i.e., limit *T*_*m*_.

The model that provides a theoretical estimate of *T*_*m*_ due to the nuclear magnetic noise and denoted as $T_{m}^{n}$ was developed elsewhere by a group of independent authors (Stamp and Tupitsyn, 2004; Warner et al., 2013). This estimate consists in an expression analog to Equation (2), where the electron dephasing rate γ_*e*_ is just replaced by a nuclear dephasing rate γ_*n*_. By applying this model to a single crystal of **1**, we subsequently conducted the calculation of $T_{m}^{n}$ for each one of the seven selected transitions in Figure 2. We considered the crystallographic positions of all magnetic nuclei inside a spherical cluster of 40 Å radius, which was enough to converge γ_*n*_. Our results show that $T_{m}^{n}$ lies inside the range 51–68 μs, whose values are two orders of magnitude above the experimental *T*_*m*_. This reveals that magnetic nuclear decoherence might not be among the decoherence mechanisms limiting *T*_*m*_.

At any temperature, MSQs can also couple to crystal vibrations and this constitutes another important decoherence mechanism, which becomes more important as thermal energy is increased (Escalera-Moreno et al., 2018). There also exists a characteristic timescale to evaluate how long information can survive in the form of quantum superposition between two energy levels before the vibration bath make it collapse. This is the so-called spin-lattice relaxation time *T*_1_ (Schweiger and Jeschke, 2001), which is also routinely determined via EPR but its estimate is hard to calculate on paper and should be addressed in a future work (Liddle and van Slageren, 2015; Escalera-Moreno et al., 2017). In this case, the out-of-plane magnetization *M*_∥_(*t*) of the sample is measured as a function of time *t* by means of inversion or saturation recovery experiments. Then, *T*_1_ is extracted by fitting the plot *M*_∥_(*t*) vs. *t* to a function proportional to 1−exp(−*t*/*T*_1_). At *T* = 6 K, the measurements at each one of the seven transitions in Figure 2 led to *T*_1_ values around 2.5 μs as reported elsewhere (Jenkins et al., 2017). Note that this timescale is close to *T*_*m*_, which suggests that lattice vibrations might be considered as another potential source of decoherence. Due to the relatively low working temperature, the vibrational states involved might be low in energy such as those from long-wavelength phonon modes and low-frequency molecular vibrations. In fact, the latter can be significantly coupled even if they are moderately populated at low temperature as found in a different molecular system (Escalera-Moreno et al., 2017). Since the spin-vibration coupling Hamiltonians are proportional to derivatives of the anisotropy parameters respect to distortion coordinates, the relevant vibrational modes to focus on may be found among those producing significant variations in these parameters. As mentioned, the computation of these evolutions involve many point calculations that can be computationally demanding and should be worked out elsewhere. Higher energy modes might play a very limited role not significant at all because of their negligible thermal population.

At first sight, the apparent rigidity of the polyoxometalate cage would prevent from a significant vibrational decoherence. While this observation could be true, we must not forget that the Gd^III^ ion is coordinated by an apical water molecule, which is also embedded in the cage but not covalently bonded to it. This could result in a certain freedom of movement that allows the vibration of this water molecule with respect to the Gd^III^ position. Thus, both independent and collective motions of Gd^III^ ion and apical water molecule could be examples of significantly coupled low-frequency vibrations. In addition, there also exists another important decohering movement concerning rotations of magnetic nuclei. For instance, it is well-known that rotation of methyl groups -CH_3_ can couple to energy levels encoding spin qubits (Zecevic et al., 1998; Eaton and Eaton, 1999; Lin et al., 2019). In case of **1**, the rotation would take place in the coordinating water molecule, whose magnetic nuclei in the form of two hydrogen atoms would couple to the Gd^III^ ion. Of course, this would deserve further investigation, since the forced removal of this apical water molecule could result in an enhanced coherence for the three qubits encoded in the energy level structure of **1**. In fact, a family of lanthanide-based POMs with a similar coordination environment but excluding the apical water molecule has recently been theoretically explored (Baldoví and Kondinski, 2018), whose Gd^III^ derivative could also offer a promising platform to design a robust single-molecule quantum processor.

## Conclusions

The successful realization of algorithms in a quantum processor will critically depend on our ability of entangling a large enough set of qubits while keeping information under processing safe from decoherence. As far as spatial organization for a proper qubit entanglement is concerned, molecular spin qubits are promising candidates by exploiting chemical tailoring. In this work, we have investigated the [Gd(H_2_O)P_5_W_30_O_110_]^12−^ POM, whose low-lying magnetic energy scheme allows defining three spin qubits and thus could be harnessed as a minimal quantum processor. Complex algorithms will require the interplay of an increasing number of close copies of **1** to allow a robust enough communication. Hence, we estimated the effect of magnetic noise on the encoded quantum information, since it becomes more important when increasing the spatial concentration of the magnetic POM. Our results reveal that there exists a noticeable presence of magnetic noise. Indeed, on one hand the estimates $T_{m}^{n}$ ~ 51–68 μs due to the magnetic nuclei bath are two orders of magnitude above the experimentally-reported phase memory times *T*_*m*_ ~ 0.5 μs, and thus this bath should play a minor role on qubit coherence. On the other hand, the estimates $T_{m}^{e}$ ~ 3–4 μs are much closer to *T*_*m*_ ~ 0.5 μs but yet are not coincident. Thus, everything suggests that the experimental *T*_*m*_ times are not only limited by this decoherence source but rather by a set of competing mechanisms where it could be included. Among these mechanisms, vibration-induced decoherence involving low-energy modes could play a major role due to the proximity between *T*_*m*_ and the experimental value *T*_1_ ~ 2.5 μs reported at *T* = 6 K. The origin of this mechanism could be found in long-wavelength crystal vibrations but especially also in the relatively-little-restricted motion of the apical water molecule coordinating the Gd^III^ ion in **1**. Moreover, this movement could be complemented with rotations of the water molecule itself, which would result in a new decoherence source due to the magnetic nature of hydrogen atom nuclei. By all means, these observations deserve a deeper research of this POM to get further insight on the role played by this coordinating water molecule as far as qubit coherence is concerned. Thus, we expect that this manuscript will encourage to undertake future investigations to test the real potential of **1**, as well as similar related coordination compounds, to become a quantum processor and how to improve its performance.

## Data Availability Statement

The datasets generated for this study are available on request to the corresponding author.

## Author Contributions

LE-M developed the model to quantify electron magnetic noise in molecular spin qubits and conducted the calculations for the system **1**. LE-M and JB contributed to write and revise the manuscript.

### Conflict of Interest

The authors declare that the research was conducted in the absence of any commercial or financial relationships that could be construed as a potential conflict of interest.
